# A Lay Sermon

**Published:** 1882-07

**Authors:** 


### A LAY SERMON.

"And withal they learn to be idle, wandering about from house to house; and not only idle, but tattlers also, and busy bodies, speaking things which they ought not."

Almost two thousand years have passed away since Paul uttered these words, but they have the same significance to-day. Idlers and tattlers still abound, and they are just as eager to stir up strife and discord among their fellow beings. Their office is to show the dark side of human nature. They are usually too adroit to slander openly, for then they might be compelled to face the truth, and these people have as great a dread of truth and honesty as their great prototype is said to have of "holy water."

The worst slander, that which sinks the deepest, and is the most baleful in its effects, is the slander that is not uttered. Against this there is no defense. You can not follow the serpent that trails his stealthy course, concealed by the grass and underbrush. You do not see his poisonous fangs, but you know the blow has been struck. It may have been only a suspicious shrug of the shoulders, a peculiar expression of the face, or an upturning of the eyes, but it was intended to convey a meaning which the cowardly wretch dare not utter. It is a stab in the dark, beyond the reach of legal redress. The ready answer to the charge is : What have I done ? What have I said ?

There is a class of idle gossips in every community—the smaller the more active—who really mean no harm, but still do a great deal of mischief. They tattle—innocently enough—about their best and dearest friends. It is often idle talk, but it is too frequently founded on envy, jealousy, and uncharitableness. They criticise the dress of their friends, or their manners, or laugh convulsively at some little mishap they have themselves experienced a thousand times. But a sensitive spirit is wounded, often made hesitating and timid for life by constantly being subjected to ridicule or reproof.

All who seek to do good should at all times and in all places, without fear or favor, set their faces firmly against these things. Forbid your children to gossip or bring you any news about your neighbors. Hush up the professional gossip—male or female—before the first slanderous sentence is finished, by a treatment so heroic and decided that a second operation will never be necessary. You will do more for the peace and quiet of that neighborhood, more for the social elevation of its people and their self-respect, than you can imagine. We have known a single individual —a lady of refinement and influence—to reform a neighborhood of gossips in a week by firmly refusing to listen to the news. How many there are who can always see a mote in every eye, but think there is no beam in their own. How many there are eager to cast the first stone, whose own lives would not bear the light of day. It is always safe to distrust such persons and to be dumb in their presence, for if one listens and approves, he is likely to become the next victim. Finally, let each individual take that same individual under his especial supervision, leaving others to do likewise.

